# Whole genome analysis unveils genetic diversity and potential virulence determinants in *Vibrio parahaemolyticus* associated with disease outbreak among cultured *Litopenaeus vannamei* (Pacific white shrimp) in India

**DOI:** 10.1080/21505594.2021.1947448

**Published:** 2021-08-20

**Authors:** Kattapuni Suresh Prithvisagar, Ballamoole Krishna Kumar, Toshio Kodama, Praveen Rai, Tetsuya Iida, Iddya Karunasagar, Indrani Karunasagar

**Affiliations:** a Nitte (Deemed to Be University), Division of Infectious Diseases, Nitte University Centre for Science Education and Research, Deralakatte, Mangaluru-Karnataka, India; b Department of Bacterial Infections, Research Institute for Microbial Diseases, Osaka University, Osaka, Japan; c Department of Bacteriology, Institute of Tropical Medicine, Nagasaki University, NagasakiJapan

**Keywords:** Vibriosis, *Vibrio parahaemolyticus*, comparative genomics, genetic diversity, virulence

## Abstract

*Vibrio parahaemolyticus* has caused widespread mortality in Indian shrimp aquaculture in recent years. However, there are insufficient genome data for the isolates from Indian shrimp vibriosis to analyze genetic diversity and track the acquisition of genetic features that could be involved in virulence and fitness. In this study, we have performed genome analysis of *V. parahaemolyticus* isolated from moribund shrimps collected from shrimp farms along coastal Karnataka, India, for better understanding of their diversity and virulence. Five newly sequenced genomes of *V. parahaemolyticus* along with 40 genomes retrieved from NCBI were subjected to comparative genome analysis. The sequenced genomes had an overall genome size of 5.2 Mb. MLST analysis and core genome phylogenomic analysis revealed considerable genetic diversity among the isolates obtained from the moribund shrimps. Interestingly, none of the *V. parahaemolyticus* isolates possessed the classical features (PirAB) of the strains associated with Acute Hepatopancreatic Necrosis Disease (AHPND). This study also revealed the presence of multiple virulence attributes, including ZOT, ACE and RTX toxins, secretion systems, and mobile genetic elements. The findings of this study provide insights into the possible transition of an environmental *V. parahaemolyticus* to emerge as pathogens of aquaculture species by increasing its virulence and host adaptation. Future studies focusing on continuous genomic surveillance of *V. parahaemolyticus* are required to study the evolution and transmission of new variants in shrimp aquaculture, as well as to design and implement biosecurity programs to prevent disease outbreaks.

## Introduction

*Vibrio parahaemolyticus* is a member of the harveyi clade that occupies various niches of the marine ecosystem [1]. This organism is associated with water, sediment, and various aquatic flora and fauna, such as plankters, invertebrates, fish, and marine mammals [1,2]. It is a well-established foodborne pathogen responsible for inflammatory gastroenteritis in humans following ingestion of contaminated seafood. Human infection is typically correlated with the production of heat-stable toxins such as thermostable direct hemolysin (TDH) and TDH-related hemolysin (TRH) coded by *tdh* and *trh* genes, respectively [3]. Outbreaks associated with seafood due to certain genogroups designated as pandemic clones of *V. parahaemolyticus* have been reported from Asia, North America, South America, and Europe [4,5]. Continued outbreaks from different parts of the world led the FAO/WHO to assess the risk associated with seafood contamination by *V. parahaemolyticus*, recommend monitoring of distribution of this organism in seafood globally, and prepare guidelines for seafood trade [2].

The shrimp aquaculture industry has expanded from 15% to 18% in the international seafood trade over a decade with an expected compound annual growth rate of 5.7% during 2017 to 2020 ([6,7] Highlights no. 1–17). However, with the intensification of aquaculture and environmental changes, diseases have become a major problem in the Asia Pacific region and Latin American countries. While most of the shrimp diseases have been due to viral agents, the disease caused by *V. parahaemolyticus* has received considerable attention from the scientific community. Some strains of this bacteria have been identified as a shrimp pathogen causing Acute Hepatopancreatic Necrosis Disease (AHPND) resulting in mass mortality in farmed shrimps in several parts of the world [4,8]. Unusual disease outbreaks due to AHPND began in China during 2009, rapidly spreading to Vietnam, Malaysia, Thailand, and Mexico [9–11]. AHPND affects shrimps in the post-larval or juvenile stage within 30–35 days post stocking and can cause near 100% mortality [12,13]. Studies have revealed that AHPND-causing *V. parahaemolyticus* possesses a unique 70kb plasmid (pVA1) encoding a binary toxin, photorhabdus insect-related (Pir) coded by *pirA* and *pirB* genes [8]. PirAB toxins induce pore formation in the hepatopancreatic cells, leading to tissue degradation and digestive dysfunction that finally results in cell necrosis [8,13]. The animals affected by AHPND are characterized by severe atrophy of the hepatopancreas with massive sloughing of epithelial cells. However, in recent years, reports of PirAB lacking *V. parahaemolyticus* infections have been reported [13,14]. AHPND has not been detected in India, but reports of *V. parahaemolyticus* strains lacking *pirA* and *pirB* genes have been associated with mortalities in farmed shrimp during 2013 [15].

Whole-genome sequence analysis of *V. parahaemolyticus* isolated worldwide has shown high genomic diversity with respect to their virulence and targeted hosts [16]. Many of the virulence attributes are acquired by horizontal gene transfer (HGT) through mobile genetic elements [17]. The genome plasticity and genetic diversity of mobile elements associated with *V. parahaemolyticus* may affect the survival of the pathogen and its infection capabilities. Also, understanding the relationship between epidemiological links among different pathogenic strains isolated worldwide and the genome complexity and diversity of *V. parahaemolyticus* [18] would be important. Recent studies have shown that environmental isolates of *V. parahaemolyticus* acquire the virulence attributes that would make them potentially pathogenic for humans and aquatic animals [12,19].

We performed whole-genome sequencing of *V. parahaemolyticus* isolated from moribund shrimps collected from shrimp farms along coastal Karnataka, India, and compared the sequences with global strains to better understand the virulence attributes and diversity of *V. parahaemolyticus*. The data would be useful to explore the pathogenesis mechanism of this opportunistic pathogen to cause pathological changes in the affected animals and the molecular factors that allow *V. parahaemolyticus* to adapt and grow in various niches.

## Results and Discussion

The intensification of the shrimp aquaculture industry, and the consequent occurrence of diseases, is a constraint to the progress of the sector. *V. parahaemolyticus*-associated disease of cultured shrimp is an emerging disease in aquaculture that has seriously impacted food productivity, animal welfare, human health, and, consequently, the country’s overall economic development. In late 2013, the first documented example of large-scale mortalities among cultured *Litopenaeus vannamei* (Pacific white shrimp) owing to *V. parahaemolyticus* were recorded in grow-out farms within 40–50 days of stocking in India, and the disease continues to be a scourge for aquaculture [15,20,21]. Surprisingly, none of the *V. parahaemolyticus* isolates obtained from the diseased shrimps exhibited the classic characteristics of the strains associated with AHPND. To investigate the genomic characteristics of the disease-causing strains, we performed whole-genome sequencing and comparative genomic analysis on *V. parahaemolyticus* associated with an outbreak among cultured *L. vannamei* in coastal Karnataka grow-out ponds.

**Figure 1.**
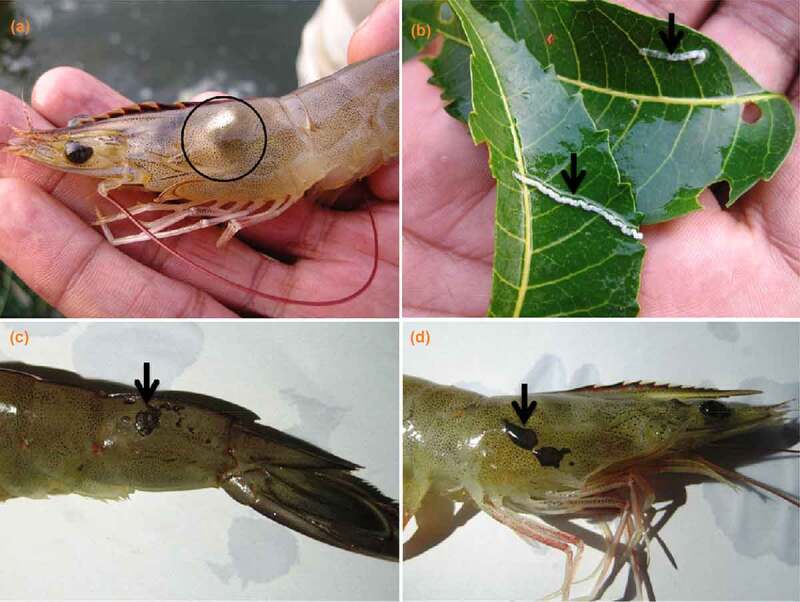
Infected *Litopenaeus vannamei* (Pacific white shrimp) from grow-out ponds of coastal Karnataka. (a) cloudy hepatopancreas (b) white feces, (c and d) brown to black spots on their exoskeleton

Severe mortalities among cultured *L. vannamei* were reported in coastal Karnataka grow-out ponds in early 2017. Infected animals exhibited erratic swimming, decreased feeding, cloudy hepatopancreas, brown to black spots on their exoskeleton, and strings of white feces floating in the water column (Figure 1). Bacteriological analysis of hemolymph, aseptically drawn using a syringe from 100 moribund shrimps from 10 ponds and plated on thiosulfate citrate bile salt (TCBS) agar yielded greenish-blue colonies. Their biochemical reactions matched the phenotypic characteristics of standard *V. parahaemolyticus* culture. Eighty isolates of *V. parahaemolyticus* obtained were genotypically confirmed by the presence of *toxR* and *tlh* genes [22,23]. As all analyzed samples were negative for the shrimp viruses tested, they are not discussed further [24]. Four representative samples originating from different ponds were selected based on the extent of shrimp mortality in the ponds, for their ability to cause infection in laboratory trials on shrimps (results not shown) and for the presence of virulence gene markers. All four isolates were negative for the *tdh, trh*, and pirAB genes, but their T6SS profiles differed with two isolates possessing both T6SSs and the other two negative for T6SS1 and positive for T6SS2. Additionally, an oyster isolate positive for the *tdh* and *trh* gene was chosen to study the genetic composition and differences, if any, from *V. parahaemolyticus* recovered from the cases of shrimp mortality, through comparative analysis.

### General genomic features and annotation of the sequenced isolates

The raw reads generated from the five *V. parahaemolyticus* isolates were evaluated for their quality and were found to be appropriate for downstream genome analysis. *de-novo* assembly of raw reads using Unicycler v0.4.8 generated high-quality draft genomes with an average coverage of 800X and >89% completeness estimated by QUAST. The *V. parahaemolyticus* isolates sequenced in this study had an overall genome size of approximately 5.2 Mb (5.09–5.35 Mbp) with an average GC content of 45%, which was similar to the reference strain RIMD2210633 (Table 1) and other *V. parahaemolyticus* genomes available in the NCBI GenBank [25–28]. The pairwise ANI percent and dDDH values revealed a high degree of sequence similarity to the reference strain RIMD2210633. The detailed information on pairwise ANI percent and dDDH values of each isolate is given in Table S1. The RAST annotation of the draft genomes revealed the presence of approximately 4,895 CDS in which an average of 3,798 proteins was assigned a functional category and 1,096 identified as hypothetical proteins (Table S2).

**Table 1.** Genome properties of *V. parahaemolyticus* sequenced in this study

**Strain**	**No. of reads (M)**	**Average read length**	**Genome length**	**No. of contigs**	**GC content (%)**	**Contig L50**	**Contig N50**
**HP1**	11.2	250	5,096,571	64	45.32	3	598,901
**NUK/7**	14.0	250	5,356,646	109	45.24	4	556,280
**VP32**	2.4	275	5,203,576	77	45.36	7	314,045
**SHP/2**	12.9	250	5,080,318	93	45.32	5	457,408
**81TDH2**	2.0	275	5,096,086	117	45.28	9	161,357

### MLST-based phylogenetic analysis

The *in silico* MLST analysis of the five *V. parahaemolyticus* isolates sequenced from this study demonstrated five different sequence types (STs) and among them, three STs were identified with previously assigned STs in the pubMLST database (https://pubmlst.org/), HP1 – ST428; SHP/2 – ST363 and 81TDH2 – ST675. The remaining two isolates, NUK/7 and VP32 showed unique MLST allelic profiles, hence was submitted to the PubMLST database to assign allele identifier number and sequence type. These two isolates were assigned with new sequence types, ST2429 for NUK/7 and ST2428 for VP32, by the pubMLST database curator (Table 2). The previous studies have identified several clonal complexes in the *V. parahaemolyticus*, including CC3, comprising pandemic strains and six clonal complexes of clinical isolates [29,30]. It is important to note that none of the STs identified in this study belongs to any of the clonal complexes described for the highly pathogenic clones of *V. parahaemolyticus* [29].

**Table 2.** Multi locus sequence typing of *V. parahaemolyticus* to assign sequence type (ST) The isolates with newly assigned ST are marked with ‘bold and underlined letters and new alleles are marked with “a star”

**Strain**	***dnaE***	***gyrB***	***recA***	***dtdS***	***pntA***	***pyrC***	***tnaA***	**ST**
**HP1**	171	222	113	126	4	62	23	428
**NUK/7**	35	499	31	19*	128	26*	226	**2429**
**VP32**	5	106	98	33*	56	96	57*	**2428**
**SHP/2**	12	180	81	19	21	11	73	363
**81TDH2**	31	316	25	274	71	73	62	675

**Figure 2.**
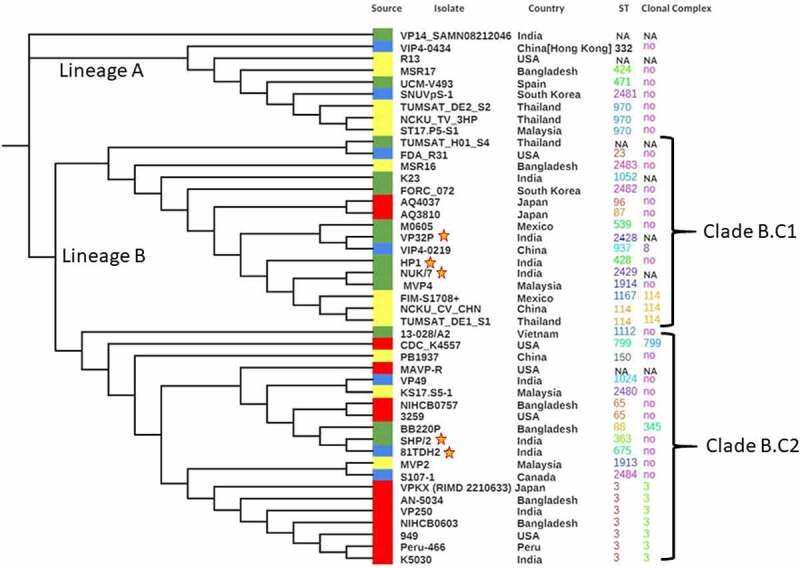
MLST-based phylogenetic analysis of clinical (red), seafood-isolated (blue), AHPND-positive (yellow), and AHPND-negative environmental (green) groups of *Vibrio parahaemolyticus* strains. The query strains are marked with “a star.”

A phylogenetic tree was constructed based on the concatenated sequences of seven housekeeping loci obtained for the five different *V. parahaemolyticus* strains sequenced in this study along with 40 global strains (retrieved from diverse sources, Table 1), whose MLST loci are available at PubMLST database (Figure 2). The resultant phylogenetic tree consisted of two major lineages, lineage A and lineage B. Surprisingly, all of the *V parahaemolyticus* isolates from vibriosis-affected *L. vannamei* in India were found scattered throughout the phylogenetic tree without restricting to a particular cluster. The results obtained in this study are in agreement with the study of Chonsin et al. [31], wherein they demonstrated that *V. parahaemolyticus* affecting *L. vannamei* were genetically diverse and not derived from a single genetic lineage. However, most of the clinical isolates included in this study showed a unique branching pattern (Clade B.C2). The overall observation from the phylogenetic analysis shows that the genome of *V. parahaemolyticus* has remarkable genetic diversity which could be due to a relatively high frequency of recombination events [29,32,33]. In conclusion, the *V. parahaemolyticus* population shows a high level of genetic diversity, which has evolved as a result of mutations and recombination events. Exposure of *V. parahaemolyticus* to changing environmental conditions could further result in the selection of new sequence types.

### Mining of virulence-associated genes and characterization of the genomic and pathogenicity islands

BLASTn and VFanalyzer were used to search for virulence genes in the genomes of the five *V. parahaemolyticus* isolates sequenced in this study. Except for 81TDH2, none of the sequenced isolates of this study possessed the classical virulence markers *(tdh* and *trh*) commonly associated with human pathogenic *V. parahaemolyticus*. Interestingly, none of the sequenced isolates possessed Pir toxin-like genes, despite the fact that four of them were isolated from Vibriosis affected shrimp from aquaculture ponds. Tran et al. [12] demonstrated that *V. parahaemolyticus* carrying Pir toxin genes were responsible for pathological changes in the hepatopancreas of AHPND – affected animals and they were negative for the *tdh* and *trh* genes. T3SS1 and T6SS2 genes were present in all five sequenced isolates, as these systems are conserved and present in both environmental and clinical isolates of *V. parahaemolyticus* [16]. It is noteworthy that T6SS1 was discovered in the genomes of *V. parahaemolyticus* NUK/7 and SHP/2, which had previously only been found in human clinical and AHPND-positive isolates [34,35]. The two loci of T6SS present in each chromosome play a role in both inter-bacterial and host–pathogen interactions [36]. The presence of T6SS among the isolates associated with disease outbreak in this study implies that acquisition of T6SS might offer a fitness advantage for this opportunistic pathogen in out-competing other microbes in the same ecological niche and thereby influencing the colonization outcomes [37]. Similarly, T3SS’s are involved in the secretion of toxins that cause cytotoxicity and enterotoxicity [25]. In contrast to T6SS, the T3SS1, known to secrete cytotoxic proteins, is present in almost all isolates, while T3SS2 is identified mainly in clinical isolates encoding tdh and/or trh genes [38,39]. Both T3SS’s play a prominent role in conferring virulence to *V. parahaemolyticus* [5,40]

VFanalyzer was used to predict 147 virulence factors related to secretion systems, toxin production, quorum sensing, iron metabolism, cellular motility, and host immune evasion genes such as adhesion and antiphagocytic factors from five sequenced genomes. The pattern of virulence profile from this study showed variation in the number of genes identified in each category, with an exception of quorum sensing and iron uptake with reference to RIMD2210633. The details of the number of genes present in each category among the five sequenced isolate and reference strain RIMD2210633 is illustrated in supplementary Figure S1. The quorum sensing genes *luxS* and *cqsA* were present in all isolates similar to reference strain. Similarly, the genes encoding for iron uptake and chemotaxis-motility showed minimum differences compared to reference strain. A capsular polysaccharide gene cluster *cpsA-Z*, which is responsible for capsular biosynthesis, was found on chromosome 2 in all of the isolates included in the study. The genes encoding capsular polysaccharides function as surface antigens, which play an important role in bacterial defense against the host immune system [41]. To establish itself within the host, a pathogen must overcome the host’s phagocytosis mechanism. All environmental isolates had a higher number of anti-phagocytosis-related genes than the reference strain. This is an intriguing finding, and more research is needed to understand the distribution and dynamics of capsular polysaccharide genes, as well as their relationship to pathogenicity and fitness in *V. parahaemolyticus* isolates. Other than NUK/7, the four sequenced isolates had ORFs encoding Type IV pilus among the adhesion factor genes. The NUK/7 genome identified two ORFs encoding Type IV pili in *Yersinia* sp. and an ORF encoding *Pseudomonas aeruginosa* LPS O antigen, identifying more genes in adhesion factors than the reference strain RIMD2210633. Adhesion factors are essential for pathogens to attach to the host, allowing the bacterium to persist in a new environment and proliferate as a symbiont or a pathogen [42]. Overall, all of the sequenced isolates possessed virulence characteristics that could allow them to colonize the host and cause disease.

**Figure 3.**
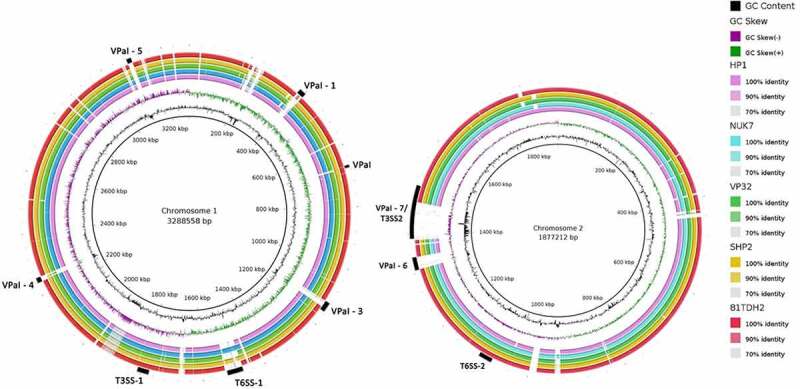
BLAST atlas for *Vibrio parahaemolyticus* chromosome 1 and 2 generated using BLAST Ring Image Generator (BRIG) against *V. parahaemolyticus* RIMD2210633 as reference genome

**Figure 4.**
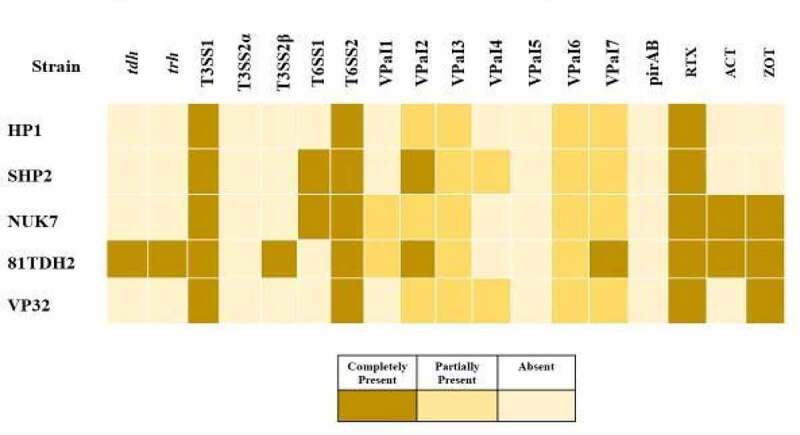
Details of virulence and toxin genes present in sequenced strains identified using annotation and BLASTn

Using the BLAST atlas tool, we compared the chromosomes of the five *V. parahaemolyticus* isolates sequenced with the reference genome of *V. parahaemolyticus* RIMD2210633 (Figure 3). The chromosome comparison revealed that the genomes had congruent gene content, variability was found in the pathogenicity island (VPaI) region and other mobile genetic elements. These pathogenicity islands on the chromosome are horizontally acquired regions flanked by repeated regions that improve the bacterium’s virulence and fitness in a specific environment [43]. Seven VPaIs have been identified in the genome of *V. parahaemolyticus* RIMD2210633 [43,44]; VPaI 5 was absent in all sequenced isolates; only SHP/2 and 81TDH2 were positive for VPaI 2; 81TDH2 harbored the complete VPaI 7 while all the other isolates had a partial sequence; partial sequences of VpaI 6 was present in all isolates; a partial island sequence of VPaI 1 in NUK/7 and 81TDH2; and a partial VPaI 4 sequence in SHP/2 and HP1 (Figure 4). BLASTn results showing a sequence with >80% sequence coverage and >95% of sequence identity to reference sequence was considered to harbor *V. parahaemolyticus* pathogenicity island (VPaI). Among the seven islands, VPaI 1, 4, and 5 are associated with the pandemic clones of *V. parahaemolyticus* while VPaI 2, 3, and 6 are completely or partially present in all isolates regardless of their pandemic potential or pathogenicity, which was evident in this study as well [4,43,45,46]. VPaI 7 harboring *tdh* and/or *trh* genes and T3SS2 cluster within its genomic region [16] is usually associated with the highly pathogenic strains of *V. parahaemolyticus*, having the ability to induce cytotoxicity and enterotoxicity and cause inflammatory gastroenteritis in humans [20,29]. This indicates that the emergence of environmental isolates with the acquisition of pathogenicity and fitness genes is a potential threat to public health [19].

### Toxins and antimicrobial resistance genes

We also identified the gene coding for ACE toxin in the chromosomes of *V. parahaemolyticus* isolates sequenced in this study (81TDH2, NUK/7), which may give them an advantage in their environment. Accessory cholera enterotoxin (ACE) is a known Ca2+ dependent symporter that aids bacteria in ion transport across the membrane encoded in the “virulence cassette” of *Vibrio cholerae* [47]. Another promising toxin encoded by the *zot* gene is zonula occludens toxin (ZOT), a secreted toxin that increases intestinal permeability and is found in many *Vibrio* spp. Among the sequenced isolates, *zot* was found in NUK/7, VP32, and 81TDH2, which may improve their pathogenicity to shrimps and humans. They are known to cause actin cytoskeleton disruption in non-toxigenic clinical *V. parahaemolyticus*, increasing the permeability and enterotoxigenic effect in host cells [47,48]. The RTX toxin was present in the genomes of all five sequenced *V. parahaemolyticus* isolates. These toxins associated with the Type I secretion system in Gram-negative bacteria have been shown to have a variety of effects, including pore formation, hemolysin, and cytotoxic activity. They are known to contribute to the virulence of several *Vibrio* species, including *V. cholerae* and *Vibrio vulnificus*. RTX toxins are reported in *V. vulnificus* and known to cause acute cytotoxicity in host cells upon contact [49]. Thadtapong et al. [50] have made similar observation wherein non-AHPND *V. parahaemolyticus* carrying ACE and ZOT along with elements of secretion system showed virulence to shrimp with the characteristic features identical to AHPND pathology. Based on these observations, we surmise that the presence of ACE, ZOT, and RTX toxin genes along with the identified secretion systems and genomic islands might have enhanced the pathogenic potential of these *V. parahaemolyticus* to cause mass mortalities in *L. vannamei.*

Bacterial resistance mechanisms are critical for their survival in the environment. Antibiotic use in the aquaculture industry has also resulted in shrimp pathogens developing resistance to the majority of commonly used antibiotics [51]. PATRIC analysis of sequenced genomes revealed an average of 74 genes responsible for antibiotic and heavy metal resistance (Figure S2). Multidrug resistance genes predominated in all the isolates. The genes responsible for resistance to acriflavine, fluoroquinolones, tetracycline, and β-lactamase were identified. In the presence of bactericidal heavy metals, environmental bacteria in the estuarine and marine environment tend to adopt mechanisms to overcome heavy metals [52]. All environmental strains tested in this study possessed resistance genes against heavy metals, such as zinc, cadmium, copper, cobalt, arsenic, and chromium, which are common heavy metals in the natural habitat where *V. parahaemolyticus* is present.

### Presence of mobile genetic elements

MOB Suite found four plasmids in NUK/7, two each in SHP/2 and VP32, and no plasmids in HP1 or 81TDH2. Plasmids in NUK/7 contained toxin genes coding for a putative insecticidal toxin from the *RhsA* superfamily, ACE, and ZOT toxins; SHP/2 contained genes for ACE, and ZOT, and VP32 had plasmid-encoded ZOT toxin genes. None of the plasmids contained the PirA and PirB binary toxin genes, which are found in *V. parahaemolyticus* strains that cause AHPND. Plasmids, as mobile genetic elements encoding toxin genes in sequenced isolates of *V. parahaemolyticus*, may contribute to improved survival in their ecological niches and to genetic diversity. Other than 81TDH2, four of the five sequenced isolates showed the presence of phage sequences in their genome (Table S3). ORFs from f237 were found among the identified prophage sequences. The phage f237 has been linked to pandemic clones since 1996, and is thought to play a key role in increasing bacterial virulence [53]. Furthermore, VP32 had prophage sequences from the Myoviridae family, while the HP1 and SHP/2 genomes contained prophage sequences from the Myoviridae and Siphoviridae families. These phages are members of the order Caudovirales, which includes marine bacteriophages known to infect *Vibrio* sp [53,54]. Apart from being acquired horizontally, some of these prophages could have integrated into the sequenced bacterial genome and may have been transferred vertically through generations [55]. Prophage sequences may be responsible for some of the genetic diversity observed in the isolates studied. CRISPR plays a crucial role in the interaction of bacteria and mobile genetic elements. Six CRISPR sequences have been identified in *V. parahaemolyticus* to date. Clinical isolates have these CRISPR sequences in their genome [56], but very few CRISPR sequences have been identified in *V. parahaemolyticus* environmental isolates. CRISPR finder identified a few potential/dubious CRISPR sequences from the sequenced genomes; however, no confirmed CRISPR sequences were found in any of our sequenced isolates (Table S3). CRISPR/Cas systems are immune genes that prevent the acquisition of mobile genetic elements in bacteria. Fu et al. [57] have shown that the absence of CRISPR/Cas elements contributes to genome plasticity due to the horizontal acquisition of new virulence and fitness genes in bacteria. In contrast to clinical isolates known to have CRISPR sequences, environmental isolates lacking CRISPR elements have a better chance of acquiring conjugative genomic elements encoding resistance or virulence genes.

### Pan and core genome phylogeny analysis

**Figure 5.**
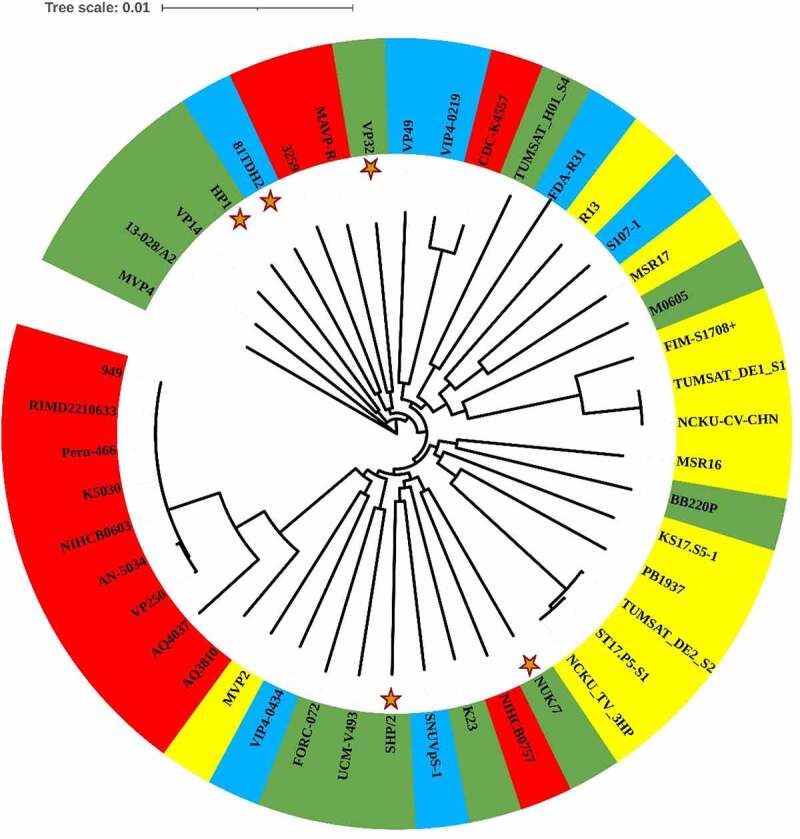
Core genome-based phylogenetic analysis of clinical (red), seafood-isolated (blue), AHPND-positive (yellow), and AHPND-negative environmental (green) groups *ofVibrio parahaemolyticus* strains. The query strains are marked with “a star.”

**Figure 6.**
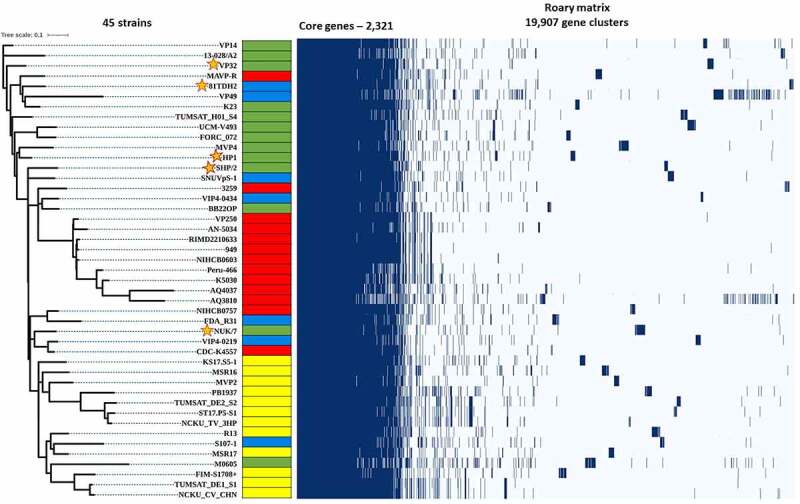
Pan genome-based phylogenetic analysis of clinical (red), seafood-isolated (blue), AHPND-positive (yellow), and AHPND-negative environmental (green) groups of *Vibrio parahaemolyticus* strains. The query strains are marked with “a star.”

The genome of five *V. parahaemolyticus* sequenced that was subjected to pan-genome analysis with 40 global isolates retrieved from NCBI GenBank using Roary revealed 19, 531 protein-coding genes. From the analysis, it is evident that as the *V. parahaemolyticus* genomes accumulated, the number of core genes reduced while the number of pan genes increased. This demonstrates the versatility of *V. parahaemolyticus* open pan-genome, which is a characteristic feature for bacteria with the capability to acquire new genes via HGT [58,59]. The open pan-genome also illustrates the diversity in the *V. parahaemolyticus* genome within the species. To determine the phylogenetic relationship of *V. parahaemolyticus* core genomes, a concatenated core genome-based phylogenetic tree was constructed using 2,321 identified core genes (Figure 5). From the core genome phylogeny, it is evident that all the newly sequenced *V. parahaemolyticus* isolates were polyphyletic; whereas, the genome of the majority of clinical isolates exhibited monophyletic branching. A pan-genome phylogenetic tree constructed, along with a gene presence-absence matrix (Figure 6), also revealed polyphyletic branching of the isolates sequenced in this study. Although the analysis is preliminary in nature, the genetic variation observed in the *V. parahaemolyticus* genome could be ecologically significant as a strategy to expand and adapt to various ecological niches as well as the driving force in the evolution of this organism [60,61]

In summary, this study unveils the genetic diversity and presence of potential virulence determinants in *V. parahaemolyticus* isolated from the disease outbreak among cultured *L. vannamei* in India. Despite the fact that these isolates were recovered from moribund shrimp and tested negative for the pVA plasmid-encoded PirAB toxin, which is associated with AHPND and shrimp virulence, their pathogenicity to the shrimp could be due to other identified toxins such as ZOT, ACE, RTX, and secretory apparatus such as T6SSs. The acquired genomic islands and mobile genetic elements detected in these isolates might be responsible for their transition from an environmental niche to an aquaculture pathogen by increasing its host adaptation fitness. The results presented in this study is limited to few isolates recovered from the disease outbreak; hence, future genomic studies involving a large number of isolates recovered from vibriosis affected shrimp and their rearing environment is necessary to understand the evolution of *V. parahaemolyticus* pathotypes and novel genetic features responsible for causing mass mortalities in culture systems. Such studies could also provide valuable information on the design and implementation of biosecurity programs to prevent disease outbreaks and develop appropriate response strategies for shrimp aquaculture management.

## Materials and Methods

### *Isolation and Identification of* V. parahaemolyticus *from moribund shrimp*

A total of 100 moribund shrimps were randomly sampled from 10 ponds where mass shrimp mortalities were observed, and bacteriological analysis was performed to identify the causative agent associated with the disease. To isolate Vibrios, hemolymph and hepatopancreas from each animal was plated on thiosulfate citrate bile salt sucrose (TCBS) agar (HiMedia Laboratories Pvt. Ltd, India). Bluish-green colonies obtained after 24 h of incubation at 30°C were subjected to a battery of biochemical tests to identify the *Vibrio* sp., as described previously [15]. Polymerase chain reaction (PCR) was used to confirm the species of the vibrio isolates by targeting the *toxR* and *tlh* genes [22,23]. All samples were also tested for the important DNA and RNA viruses affecting shrimp aquaculture in Asia by PCR as described in the OIE Aquatic Manual [24].

### Whole-genome sequencing and assembly

Whole-genome sequencing and comparative genomic analysis were performed on four confirmed cultures of *V. parahaemolyticus* isolated from moribund shrimp and one isolate from seafood (oyster) harvested from India’s southwest coast. All isolates were grown overnight at 30 °C in Luria Bertani (LB) broth supplemented with 3% NaCl and the genomic DNA was extracted by the cetyl trimethyl ammonium bromide (CTAB)-proteinase K method. Whole-genome sequencing library of genomic DNA was prepared using the Nextera XT kit (Illumina). The Illumina MiSeq platform was used to prepare the sequencing libraries. The quality of the raw reads was assessed using FastQC v0.11.8 and MultiQC v1.6 [62], and contamination was detected using Kraken v2 [63]. Trim Galore v0.6.2 [64] was used to perform low-quality base calling and adapter sequence trimming. The trimmed paired reads were subjected to *de-novo* assembly using Unicycler v0.4.8 [65], with output files generated in FASTA format. QUAST [66] was used to evaluate the quality of the assembled genomes. MOB-Suite v1.4.9 [67] was used to separate genomic and plasmid DNA. The genomic DNA contigs were reduced to single scaffolds as chromosomes 1 and 2 using MeDuSa v2/1.6 [68]. In addition, genome sequences of 40 *V. parahaemolyticus* isolates available in the NCBI database (Table 3) based on parameters, such as their global presence, source, and isolate characteristics (AHPND positive from diseased shrimp, clinical, seafood borne, environmental isolates) were chosen for downstream comparative genome analysis.

**Table 3.** Details of *V. parahaemolyticus* genome downloaded from NCBI database for comparative genome analysis

**Sl. No.**	**Strain**	**Country**	**Year**	**Source**	**Accession No.**
1	RIMD2210633	Japan	1996	Clinical (Stool)	GCA_000196095
2	CDC-K4557	USA	2007	Clinical (Stool)	GCA_000430425
3	VP250	India	1998	Clinical (Stool)	AVOK00000000
4	3259	USA	2007	Clinical (Stool)	AVOL00000000
5	NIHCB0705	Bangladesh	2006	Clinical (Stool)	AVPX00000000
6	BB22OP	Bangladesh	1982	Environmental	GCA_000328405
7	FDA-R31	USA	2007	Oyster	GCA_000430405
8	NIHCB0603	Bangladesh	2006	Clinical (Stool)	AVOM00000000
9	949	USA	2006	Consumed contaminated oyster	AVPV00000000
10	UCM-V493	Spain	2002	Sediment	GCA_000568495
11	VP49	India	2008	Seafood (Oyster)	JEMS00000000
12	Peru 466	Peru	1996	Clinical	ACFM00000000
13	SNUVpS-1	South Korea	2009	Seafood	AMRZ00000000
14	K5030	India	2005	Clinical	ACKB00000000
15	K23	India	2013	Environmental	LQGU00000000
16	AN5034	Bangladesh	1998	Clinical	ACFO00000000
17	AQ4037	Maldives	1985	Clinical	ACFN00000000
18	AQ3810	Singapore	1983	Clinical	AAWQ01000006
19	FORC_072	South Korea	2017	Environmental	GCA_003612695
20	MO605	Mexico	2013	Pacific white shrimp	JALL00000000
21	TUMSAT-DE1-S1	Thailand	-	AHPND shrimp	BAVF01000000
22	TUMSAT-DE2-S2	Thailand	-	AHPND shrimp	BAVG01000000
23	13–028/A2	Vietnam	2013	Shrimp (*L. vannamei*)	MWVH00000000
24	MSR16	Bangladesh	2016	*Penaeus monodon*	RPDA00000000
25	MSR17	Bangladesh	2017	*P. monodon*	RPDB00000000
26	TUMSAT-HO1-S4	Thailand	-	Shrimp farm	BAVI01000000
27	NCKU-CV-CHN	China	2010	Shrimp pond	JPKU00000000
28	VP14	India	2014	*L. vannamei*	PKMB00000000
29	MVp2	Malaysia	2016	Aquaculture pond	MSBY01000000
30	MVp4	Malaysia	2016	Aquaculture pond	MSBZ01000000
31	PB1937	China	2012	Shrimp	GCA_003351885
32	FIM-S1708^+^	Mexico	2014	Sediment	JPLV00000000
33	ST-17.P5-S1	Malaysia	2017	*L. vannamei*	PJOR00000000
34	KS-17.S5-1	Malaysia	2017	*L. vannamei*	PJJY00000000
35	VIP4-0219	Hong Kong	2006	Seafood	AXNQ00000000
36	VIP4-0434	Hong Kong	2008	Seafood	AXNN00000000
37	NCKU-TV-3HP	Thailand	1999	AHPND (*L. vannamei* hepatopancreas)	JPKS00000000
38	R13	USA	2016	*L. vannamei*	GCA_003119375
39	MAVP-R	USA	2011	clinical	GCA_002220985
40	S107-1	Canada	2005	Oyster	GCA_003047085
41	81TDH2	India	2003	Oyster	This study
42	VP32	India	2016	Diseased shrimp (*L. vannamei)*	This study
43	SHP/2	India	2017	Diseased shrimp (*L. vannamei)*	This study
44	HP1	India	2017	Diseased shrimp (*L. vannamei)*	This study
45	NUK/7	India	2017	Diseased shrimp (*L. vannamei)*	This study

### In-silico *taxonomic validation and genome annotation*

The species identity was confirmed using JSpecies v1.2.1 [69] to calculate the pairwise average nucleotide identity (ANI) of the assembled genomes with *V. parahaemolyticus* RIMD2210633. In addition, the GGDC 2.0 server [70] was used to calculate digital DNA–DNA hybridization (dDDH). The Rapid Annotations using Subsystems Technology (RAST) server [71] was used to perform structural gene prediction and functional annotations. Using the BLAST Ring Image Generator (BRIG) [72], the annotated chromosomes were visualized in the BLAST atlas. Phigaro v2.2.0 [73] helped detect the presence of putative prophage sequences in assembled genomes. VFanalyzer [74] and NCBI BLASTn were used to compare the presence of putative virulence factors with *V. parahaemolyticus* RIMD2210633. To predict the presence of antibiotic resistance genes, PATRIC and the CARD web portal were used and CRISPRfinder [75] served to annotate the CRISPR elements in the *V. parahaemolyticus* sequenced genomes.

### MLST and phylogenetic analysis

The stringMLST tool [76] helped perform *in-silico* MLST analysis on the sequenced strains. Furthermore, a neighbor-joining phylogenetic tree calculated with default parameters from concatenated sequence alignments of seven housekeeping genes from five sequenced isolates and 40 global sequences was constructed using iTOL v3 [77] for the MLST-based phylogenetic analysis.

### Pan genome analysis

Roary v3.13.0 [78] was employed for the pan-genome analysis. To generate gff3 files, 40 NCBI GenBank reference strains and 5 newly sequenced strains were annotated using Prokka v1.13.4 [79]. Thereafter, the annotated files were subjected to pan genome analysis with a minimum BLASTP identity cutoff of 95%. The genes present in 99% of the total isolates were identified as core genes. The resulting core genome alignment was then used to build a core genome-based phylogenetic tree with IQ-TREE v 1.5.5.3 [80] with 1000 bootstrap replicates and visualized with iTOL v3 [77]. Similarly, a pan-genome-based phylogenetic tree was constructed and visualized with the gene presence-absence profile of the 45 isolates analyzed.

## Supplementary Material

Supplemental Material

## Data Availability

The whole-genome sequence data of the isolates 81TDH2 (CP066244, CP066245), HP1 (CP069236, CP069237), SHP/2 (CP066156-CP066159), NUK/7 (CP066160-CP066165) and VP32 (JABFAH010000000) have been assigned GenBank accession numbers following submission. The new sequence alleles identified and new MLST profiles of NUK/7 (id – 3424) and VP32 (id – 3426, isolate name VP32P in database) have been submitted to pubMLST database (https://pubmlst.org).

## References

[1] Nair GB, Ramamurthy T, Bhattacharya SK, et al. Global dissemination of *Vibrio parahaemolyticus* serotype O3: K6 and its serovariants. Clin Microbiol Rev. 2007 Jan 1;20(1):39–48. 17223622 10.1128/CMR.00025-06 PMC1797631

[2] Karunasagar I, Karunasagar I. Ecology, virulence factors and global spread of *Vibrio parahaemolyticus*. Asian Fish Sci. 2018;31:15–28.

[3] Nishibuchi M, Kaper JB. Thermostable direct hemolysin gene of *Vibrio parahaemolyticus*: a virulence gene acquired by a marine bacterium. Infect Immun. 1995 Jun;63(6):2093. 7768586 10.1128/iai.63.6.2093-2099.1995 PMC173271

[4] Ceccarelli D, Hasan NA, Huq A, et al. Distribution and dynamics of epidemic and pandemic *Vibrio parahaemolyticus* virulence factors. Front Cell Infect Microbiol. 2013 Dec 11;3: 97. 24377090 10.3389/fcimb.2013.00097 PMC3858888

[5] Karunasagar I, Kumar KB, Nair GB. Epidemiology and genetics of the pandemic clone of *Vibrio parahaemoluyicus*. In: Foodborne and Waterborne Bacterial Pathogens Epidemiology, Evolution and Molecular Biology. Norfolk (UK): Caister Academic Press; 2012. p. 185–196. DOI:10.21775/9781912530007

[6] Food and Agriculture Organization of the United Nations. FAO Yearbook, Fishery and Aquaculture Statistics, 2011.

[7] FAO. GLOBEFISH Highlights January 2020 ISSUE, with Jan. – Sep. 2019 Statistics – A quarterly update on world seafood markets. Globefish Highlights no. 1–2020. Rome.

[8] Lee CT, Chen IT, Yang YT, et al. The opportunistic marine pathogen *Vibrio parahaemolyticus* becomes virulent by acquiring a plasmid that expresses a deadly toxin. Proc Natl Acad Sci U S A. 2015 Aug 25;112(34):10798–10803. 10.1073/pnas.1503129112 PMC4553777 26261348

[9] Hong X, Lu L, Xu D. Progress in research on acute hepatopancreatic necrosis disease (AHPND). Aquac Int. 2016 Apr 1;24(2):577–593.

[10] Yan CZ, Austin CM, Ayub Q, et al. Genomic characterization of *Vibrio parahaemolyticus* from Pacific white shrimp and rearing water in Malaysia reveals novel sequence types and structural variation in genomic regions containing the Photorhabdus insect-related (Pir) toxin-like genes. FEMS Microbiol Lett. 2019 Sep;366(17):fnz211. 31589302 10.1093/femsle/fnz211

[11] Wang HC, Lin SJ, Mohapatra A, et al. A review of the functional annotations of important genes in the AHPND-causing pVA1 plasmid. Microorganisms. 2020 Jul;8(7):996. 10.3390/microorganisms8070996 PMC7409025 32635298

[12] Tran L, Nunan L, Redman RM, et al. Determination of the infectious nature of the agent of acute hepatopancreatic necrosis syndrome affecting penaeid shrimp. Dis Aquat Organ. 2013 Jul 9;105(1):45–55. 23836769 10.3354/dao02621

[13] Joshi J, Srisala J, Truong VH, et al. Variation in *Vibrio parahaemolyticus* isolates from a single Thai shrimp farm experiencing an outbreak of acute hepatopancreatic necrosis disease (AHPND). Aquaculture. 2014 May 20;428: 297–302.

[14] Zhang X, Sun J, Chen F, et al. Phenotypic and genomic characterization of a Vibrio parahaemolyticus strain causing disease in *Penaeus vannamei* provides insights into its niche adaptation and pathogenic mechanism. Microb Genom. 2021 May 5;7(5):000549. 10.1099/mgen.0.000549 PMC8209731 33952389

[15] Kumar BK, Deekshit VK, Raj JR, et al. Diversity of *Vibrio parahaemolyticus* associated with disease outbreak among cultured *Litopenaeus vannamei* (Pacific white shrimp) in India. Aquaculture. 2014b Sep 20;433: 247–251.

[16] Ronholm J, Petronella N, Leung CC, et al. Genomic features of environmental and clinical *Vibrio parahaemolyticus* isolates lacking recognized virulence factors are dissimilar. Appl Environ Microbiol. 2016 Feb 15;82(4):1102–1113. 26637607 10.1128/AEM.03465-15 PMC4751838

[17] Deng Y, Xu H, Su Y, et al. Horizontal gene transfer contributes to virulence and antibiotic resistance of *Vibrio harveyi* 345 based on complete genome sequence analysis. BMC Genomics. 2019 Dec 1;20(1):761. 31640552 10.1186/s12864-019-6137-8 PMC6805501

[18] Fu S, Tian H, Wei D, et al. Delineating the origins of *Vibrio parahaemolyticus* isolated from outbreaks of acute hepatopancreatic necrosis disease in Asia by the use of whole genome sequencing. Front Microbiol. 2017 Nov 28;8: 2354. 29234316 10.3389/fmicb.2017.02354 PMC5712426

[19] Gennari M, Ghidini V, Caburlotto G, et al. Virulence genes and pathogenicity islands in environmental *Vibrio* strains nonpathogenic to humans. FEMS Microbiol Ecol. 2012 Dec 1;82(3):563–573. 22676367 10.1111/j.1574-6941.2012.01427.x

[20] Sanathkumar H, Ravi C, Padinhatupurayil SB, et al. Microbiological investigation of persistent mortalities in *Litopenaeus vannamei* grown in low saline waters in India. J Aquat Anim Health. 2014 Jul 3;26(3):154–159. 25229486 10.1080/08997659.2014.902875

[21] Navaneeth KA, Bhuvaneswari T, Rajan JJ, et al. Characterization of *Vibrio parahaemolyticus* isolates from shrimp farms of Southeast coast of India with special reference to Acute Hepatopancreatic Necrosis Disease (AHPND) status. Aquaculture. 2020 Mar 15;518: 734813.

[22] Bej AK, Patterson DP, Brasher CW, et al. Detection of total and hemolysin-producing *Vibrio parahaemolyticus* in shellfish using multiplex PCR amplification of tl, tdh and trh. J Microbiol Methods. 1999 Jun 1;36(3):215–225. 10379807 10.1016/s0167-7012(99)00037-8

[23] Kim YB, Okuda JU, Matsumoto C, et al. Identification of *Vibrio parahaemolyticus* strains at the species level by PCR targeted to the toxR gene. J Clin Microbiol. 1999 Apr 1;37(4):1173–1177. 10074546 10.1128/jcm.37.4.1173-1177.1999 PMC88669

[24] International Office of Epizootics. Aquatic Animal Health Standards Commission. Manual of diagnostic tests for aquatic animals. Office international des épizooties; France: 2019.

[25] Makino K, Oshima K, Kurokawa K, et al. Genome sequence of *Vibrio parahaemolyticus*: a pathogenic mechanism distinct from that of *V. cholerae*. Lancet. 2003 Mar 1;361(9359):743–749. 12620739 10.1016/S0140-6736(03)12659-1

[26] Liu M, Chen S. Draft genome sequence of *Vibrio parahaemolyticus* V110, isolated from shrimp in Hong Kong. Genome Announc. 2013 Jun 27;1(3):3. 10.1128/genomeA.00300-13 PMC3707586 23788537

[27] Gomez-Gil B, Soto-Rodríguez S, Lozano R, et al. Draft genome sequence of *Vibrio parahaemolyticus* strain M0605, which causes severe mortalities of shrimps in Mexico. Genome Announc. 2014 May 1;2(2):2. 10.1128/genomeA.00055-14 PMC3945492 24604636

[28] Kumar BK, Deekshit VK, Rai P, et al. Draft Genome Sequence of *trh*+*Vibrio parahaemolyticus* VP-49, Isolated from Seafood Harvested along the Mangalore Coast, India. Genome Announc. 2014a Jun 26;2(3):3. 10.1128/genomeA.00607-14 PMC4073111 24970827

[29] González-Escalona N, Martinez-Urtaza J, Romero J, et al. Determination of molecular phylogenetics of *Vibrio parahaemolyticus* strains by multilocus sequence typing. J Bacteriol. 2008 Apr 15;190(8):2831–2840. 18281404 10.1128/JB.01808-07 PMC2293261

[30] Han D, Tang H, Ren C, et al. Prevalence and genetic diversity of clinical *Vibrio parahaemolyticus* isolates from China, revealed by multilocus sequence typing scheme. Front Microbiol. 2015 Apr 9;6: 291. 25914691 10.3389/fmicb.2015.00291 PMC4391058

[31] Chonsin K, Matsuda S, Theethakaew C, et al. Genetic diversity of *Vibrio parahaemolyticus* strains isolated from farmed Pacific white shrimp and ambient pond water affected by acute hepatopancreatic necrosis disease outbreak in Thailand. FEMS Microbiol Lett. 2016 Jan;363(2):fnv222. 10.1093/femsle/fnv222 26590959

[32] Theethakaew C, Feil EJ, Castillo-Ramï S, et al. Genetic relationships of *Vibrio parahaemolyticus* isolates from clinical, human carrier, and environmental sources in Thailand, determined by multilocus sequence analysis. Appl Environ Microbiol. 2013 Apr 1;79(7):2358–2370. 23377932 10.1128/AEM.03067-12 PMC3623249

[33] Gonzalez-Escalona N, Jolley KA, Reed E, et al. Defining a core genome multilocus sequence typing scheme for the global epidemiology of *Vibrio parahaemolyticus*. J Clin Microbiol. 2017 Jun 1;55(6):1682–1697. 28330888 10.1128/JCM.00227-17 PMC5442524

[34] Yu Y, Yang H, Li J, et al. Putative type VI secretion systems of *Vibrio parahaemolyticus* contribute to adhesion to cultured cell monolayers. Arch Microbiol. 2012 Oct 1;194(10):827–835. 22535222 10.1007/s00203-012-0816-z

[35] Li H, Tang R, Lou Y, et al. A comprehensive epidemiological research for clinical *Vibrio parahaemolyticus* in Shanghai. Front Microbiol. 2017 Jun 8;8: 1043. 28642752 10.3389/fmicb.2017.01043 PMC5462930

[36] Salomon D, Gonzalez H, Updegraff BL, et al. *Vibrio parahaemolyticus* type VI secretion system 1 is activated in marine conditions to target bacteria, and is differentially regulated from system 2. PloS One. 2013 Apr 16;8(4):e61086. 23613791 10.1371/journal.pone.0061086 PMC3628861

[37] Yang Q, Dong X, Xie G, et al. Comparative genomic analysis unravels the transmission pattern and intra-species divergence of acute hepatopancreatic necrosis disease (AHPND)-causing *Vibrio parahaemolyticus* strains. Mol Genet Genomics. 2019 Aug; 294(4):1007–1022. 30968246 10.1007/s00438-019-01559-7

[38] Kodama T, Hiyoshi H, Gotoh K, et al. Identification of two translocon proteins of *Vibrio parahaemolyticus* type III secretion system 2. Infect Immun. 2008 Sep;76(9):4282. 18541652 10.1128/IAI.01738-07 PMC2519421

[39] Okada N, Iida T, Park KS, et al. Identification and characterization of a novel type III secretion system in *trh*-positive *Vibrio parahaemolyticus* strain TH3996 reveal genetic lineage and diversity of pathogenic machinery beyond the species level. Infect Immun. 2009 Feb;77(2):904. 19075025 10.1128/IAI.01184-08 PMC2632016

[40] Broberg CA, Calder TJ, Orth K. *Vibrio parahaemolyticus* cell biology and pathogenicity determinants. Microbes Infect. 2011 Nov 1;13(12–13):992–1001. 21782964 10.1016/j.micinf.2011.06.013 PMC3384537

[41] Chen Y, Dai J, Morris JG, et al. Genetic analysis of the capsule polysaccharide (K antigen) and exopolysaccharide genes in pandemic *Vibrio parahaemolyticus* O3:K6. BMC Microbiol. 2010 Dec 1;10(1):274. 21044320 10.1186/1471-2180-10-274 PMC2987987

[42] Stones DH, Krachler AM, Paniagua-Contreras GL. Against the tide: the role of bacterial adhesion in host colonization. Biochem Soc Trans. 2016 Dec 15;44(6):1571–1580. 27913666 10.1042/BST20160186 PMC5134996

[43] Hurley CC, Quirke A, Reen FJ, et al. Four genomic islands that mark post-1995 pandemic *Vibrio parahaemolyticus* isolates. BMC Genomics. 2006 Dec 1;7(1):104. 16672049 10.1186/1471-2164-7-104 PMC1464126

[44] Boyd EF, Cohen AL, Naughton LM, et al. Molecular analysis of the emergence of pandemic *Vibrio parahaemolyticus*. BMC Microbiol. 2008 Dec 1;8(1):110. 18590559 10.1186/1471-2180-8-110 PMC2491623

[45] Chao G, Jiao X, Zhou X, et al. Systematic functional pandemic strain–specific genes, three genomic islands, two t3sss in foodborne, and clinical *Vibrio parahaemolyticus* isolates in China. Foodborne Pathog Dis. 2009 Jul 1;6(6):689–698. 19425827 10.1089/fpd.2009.0274

[46] Chao G, Jiao X, Zhou X, et al. Distribution of genes encoding four pathogenicity islands (VPaIs), T6SS, biofilm, and type I pilus in food and clinical strains of *Vibrio parahaemolyticus* in China. Foodborne Pathog Dis. 2010 Jun 1;7(6):649–658. 20132020 10.1089/fpd.2009.0441

[47] Pérez-Reytor D, Jaña V, Pavez L, et al. Accessory toxins of *Vibrio* pathogens and their role in epithelial disruption during infection. Front Microbiol. 2018 Sep 20;9: 2248. 30294318 10.3389/fmicb.2018.02248 PMC6158335

[48] Pérez-Reytor D, Pavón A, Lopez-Joven C, et al. Analysis of the Zonula occludens Toxin Found in the Genome of the Chilean Non-toxigenic *Vibrio parahaemolyticus* Strain PMC53.7. Front Cell Infect Microbiol. 2020 Sep 24;10: 482. 33072618 10.3389/fcimb.2020.00482 PMC7541967

[49] Kim YR, Lee SE, Kook H, et al. *Vibrio vulnificus* RTX toxin kills host cells only after contact of the bacteria with host cells. Cell Microbiol. 2008 Apr;10(4):848–862. 18005241 10.1111/j.1462-5822.2007.01088.x

[50] Thadtapong N, Salinas MBS, Charoensawan V, et al. Genome Characterization and Comparison of Early Mortality Syndrome Causing *Vibrio parahaemolyticus* pirABvp− Mutant from Thailand with *V. parahaemolyticus* pirABvp+ AHPND isolates. Front Mar Sci. 2020 Apr 28;7:290.

[51] Jo S, Shin C, Shin Y, et al. Heavy metal and antibiotic co-resistance in *Vibrio parahaemolyticus* isolated from shellfish. Mar Pollut Bull. 2020 Jul 1;156: 111246. 32510388 10.1016/j.marpolbul.2020.111246

[52] Malik A, Aleem A. Incidence of metal and antibiotic resistance in *Pseudomonas* spp. from the river water, agricultural soil irrigated with wastewater and groundwater. Environ Monit Assess. 2011 Jul 1;178(1–4):293–308. 20853188 10.1007/s10661-010-1690-2

[53] Nasu H, Iida T, Sugahara T, et al. Associated with Recent Pandemic *Vibrio parahaemolyticus* O3: K6 Strains. J Clin Microbiol. 2000 Jun 1;38(6):2156–2161. 10834969 10.1128/jcm.38.6.2156-2161.2000 PMC86752

[54] Kim JH, Jun JW, Choresca CH, et al. Complete genome sequence of a novel marine siphovirus, pVp-1, infecting *Vibrio parahaemolyticus*. J Virol. 2012;7013–7014. DOI:10.1128/JVI.00742-12. PMC3393574 22628398

[55] Bobay LM, Touchon M, Rocha EP Pervasive domestication of defective prophages by bacteria. Proc Natl Acad Sci U S A. 2014 Aug 19;111(33):12127–12132. 10.1073/pnas.1405336111 PMC4143005 25092302

[56] Li L, Wong HC, Nong W, et al. Comparative genomic analysis of clinical and environmental strains provides insight into the pathogenicity and evolution of *Vibrio parahaemolyticus*. BMC Genomics. 2014 Dec 1;15(1):1135. 25518728 10.1186/1471-2164-15-1135 PMC4320434

[57] Fu S, Wang L, Tian H, et al. Pathogenicity and genomic characterization of *Vibrio parahaemolyticus* strain PB1937 causing shrimp acute hepatopancreatic necrosis disease in China. Ann Microbiol. 2018 Apr;68(4):175–184.

[58] Castillo D, Pérez-Reytor D, Plaza N, et al. Exploring the genomic traits of non-toxigenic *Vibrio parahaemolyticus* strains isolated in Southern Chile. Front Microbiol. 2018 Feb;8(9):161. 10.3389/fmicb.2018.00161 PMC5809470 29472910

[59] Paniagua-Contreras GL, Monroy-Pérez E, Díaz-Velásquez CE, et al. Whole-genome sequence analysis of multidrug-resistant uropathogenic strains of *Escherichia coli* from Mexico. Infect Drug Resist. 2019;12:2363. 31447566 10.2147/IDR.S203661 PMC6682767

[60] Cui Y, Yang C, Qiu H, et al. The landscape of coadaptation in *Vibrio parahaemolyticus*. Elife. 2020 Mar;20(9):e54136. 10.7554/eLife.54136 PMC7101233 32195663

[61] Pant A, Bag S, Saha B, et al. Molecular insights into the genome dynamics and interactions between core and acquired genomes of *Vibrio cholerae*. Proc Natl Acad Sci U S A. 2020 Sep 22;117(38):23762–23773. 10.1073/pnas.2006283117 PMC7519391 32873641

[62] Ewels P, Magnusson M, Lundin S, et al. MultiQC: summarize analysis results for multiple tools and samples in a single report. Bioinformatics. 2016 Oct 1;32(19):3047–3048. 27312411 10.1093/bioinformatics/btw354 PMC5039924

[63] Wood DE, Salzberg SL. Kraken: ultrafast metagenomic sequence classification using exact alignments. Genome Biol. 2014 Mar;15(3):1–2. 10.1186/gb-2014-15-3-r46 PMC4053813 24580807

[64] Martin M. Cutadapt removes adapter sequences from high-throughput sequencing reads. EMBnet. 2011 May 2;17(1):10–12.

[65] Wick RR, Judd LM, Gorrie CL, et al. Unicycler: resolving bacterial genome assemblies from short and long sequencing reads. PLoS Comput Biol. 2017 Jun 8;13(6):e1005595. 28594827 10.1371/journal.pcbi.1005595 PMC5481147

[66] Gurevich A, Saveliev V, Vyahhi N, et al. QUAST: quality assessment tool for genome assemblies. Bioinformatics. 2013 Apr 15;29(8):1072–1075. 23422339 10.1093/bioinformatics/btt086 PMC3624806

[67] Robertson J, Nash JH. MOB-suite: software tools for clustering, reconstruction and typing of plasmids from draft assemblies. Microb Genom. 2018 Aug;4(8):8. 10.1099/mgen.0.000206 PMC6159552 30052170

[68] Bosi E, Donati B, Galardini M, et al. MeDuSa: a multi-draft-based scaffolder. Bioinformatics. 2015 Aug 1;31(15):2443–2451. 25810435 10.1093/bioinformatics/btv171

[69] Richter M, Rosselló-Móra R, Oliver Glöckner F, et al. JSpeciesWS: a web server for prokaryotic species circumscription based on pairwise genome comparison. Bioinformatics. 2016 Mar 15;32(6):929–931. 26576653 10.1093/bioinformatics/btv681 PMC5939971

[70] Meier-Kolthoff JP, Auch AF, Klenk HP, et al. Genome sequence-based species delimitation with confidence intervals and improved distance functions. BMC Bioinformatics. 2013 Dec 1;14(1):60. 23432962 10.1186/1471-2105-14-60 PMC3665452

[71] Aziz RK, Bartels D, Best AA, et al. RAST Server: rapid annotations using subsystems technology. BMC Genomics. 2008 Dec;9(1):1–5. 18261238 10.1186/1471-2164-9-75 PMC2265698

[72] Alikhan NF, Petty NK, Zakour NL, et al. BLAST Ring Image Generator (BRIG): simple prokaryote genome comparisons. BMC Genomics. 2011 Dec 1;12(1):402. 21824423 10.1186/1471-2164-12-402 PMC3163573

[73] Starikova EV, Tikhonova PO, Prianichnikov NA, et al. Phigaro: high-throughput prophage sequence annotation. Bioinformatics. 2020 Jun 1;36(12):3882–3884. 32311023 10.1093/bioinformatics/btaa250

[74] Liu B, Zheng D, Jin Q, et al. VFDB 2019 : a comparative pathogenomic platform with an interactive web interface. Nucleic Acids Res. 2019 Jan 8;47(D1):D687–92. 30395255 10.1093/nar/gky1080 PMC6324032

[75] Grissa I, Vergnaud G, Pourcel C. CRISPRFinder: a web tool to identify clustered regularly interspaced short palindromic repeats. Nucleic Acids Res. 2007 Jul 1;35(suppl_2):W52–7. 17537822 10.1093/nar/gkm360 PMC1933234

[76] Gupta A, Jordan IK, Rishishwar L. stringMLST: a fast k-mer based tool for multilocus sequence typing. Bioinformatics. 2017 Jan 1;33(1):119–121. 27605103 10.1093/bioinformatics/btw586

[77] Letunic I, Bork P. Interactive tree of life (iTOL) v3: an online tool for the display and annotation of phylogenetic and other trees. Nucleic Acids Res. 2016 Apr 19;44(W1):W242–5. 27095192 10.1093/nar/gkw290 PMC4987883

[78] Page AJ, Cummins CA, Hunt M, et al. Roary: rapid large-scale prokaryote pan genome analysis. Bioinformatics. 2015 Nov 15;31(22):3691–3693. 26198102 10.1093/bioinformatics/btv421 PMC4817141

[79] Seemann T. Prokka: rapid prokaryotic genome annotation. Bioinformatics. 2014 Jul 15;30(14):2068–2069. 24642063 10.1093/bioinformatics/btu153

[80] Nguyen LT, Schmidt HA, Von Haeseler A, et al. IQ-TREE: a fast and effective stochastic algorithm for estimating maximum-likelihood phylogenies. Mol Biol Evol. 2015 Jan 1;32(1):268–274. 25371430 10.1093/molbev/msu300 PMC4271533

