# Supplementary material for: Whole genome analysis unveils genetic diversity and potential virulence determinants in Vibrio parahaemolyticus associated with disease outbreak among cultured Litopenaeus vannamei (Pacific white shrimp) in India
Source: Virulence. 2021 Aug 20;12(1):1936–49. doi: 10.1080/21505594.2021.1947448 (PMC8381830; doi:10.1080/21505594.2021.1947448)
Supplement: Supplemental Material [file KVIR_A_1947448_SM6715.zip › supplementary/Supplementary fileclean.docx]

**Whole genome analysis unveils genetic diversity and potential virulence determinants in *Vibrio parahaemolyticus* associated with disease outbreak among cultured *Litopenaeus vannamei* (Pacific white shrimp) in India**

**Kattapuni Suresh Prithvisagar^1^, Ballamoole Krishna Kumar^1*^, Toshio Kodama^2,3^, Praveen Rai^1^, Tetsuya Iida^2^, Iddya Karunasagar^1^, Indrani Karunasagar^1^,**

^1^Division of Infectious Diseases, Nitte University Centre for Science Education and Research, Nitte (Deemed to be University), Deralakatte, Mangaluru-575018, Karnataka, India

^2^Department of Bacterial Infections, Research Institute for Microbial Diseases, Osaka University, Osaka, Japan

^3^Department of Bacteriology, Institute of Tropical Medicine, Nagasaki University, Nagasaki- 853 8523, Japan

**Address for correspondence**

Ballamoole Krishna Kumar

Assistant Professor

Division of Infectious Diseases

Nitte University Centre for Science Education and Research

Nitte University, Mangaluru-575018, India

Ph: +91-824-2204292

Email: [krishnakumarb@nitte.edu.in](mailto:krishnakumarb@nitte.edu.in)

**Table S1: Average nucleotide identity (ANI) and digital DNA-DNA hybridization (dDDH) values between study genomes and reference strain *Vibrio parahaemolyticus* RIMD2210633**

|  | **RIMD2210633** | |
| --- | --- | --- |
| **Genome** | **ANI (%)** | **dDDH (%)** |
| **HP1** | 98.27 | 86.4 |
| **NUK/7** | 98.35 | 86.5 |
| **VP32** | 98.26 | 86.3 |
| **SHP/2** | 98.41 | 86.8 |
| **81TDH2** | 98.15 | 86.3 |

**Table S2: Annotation summary of the strains sequenced**

|  | **RAST Annotation** | | | **Details of the protein encoding genes using PATRIC server** | | | |
| --- | --- | --- | --- | --- | --- | --- | --- |
| **Strain** | **CDS** | **tRNA** | **rRNA** | **Proteins with functional category** | **Hypothetical proteins** | **Proteins with EC number** | **Proteins assigned to KEGG pathway** |
| **HP1** | 4,757 | 89 | 5 | 3,763 | 994 | 1,114 | 817 |
| **NUK/7** | 5,168 | 107 | 3 | 3,841 | 1,327 | 1,093 | 801 |
| **VP32** | 4,923 | 80 | 3 | 3,822 | 1,101 | 1,121 | 822 |
| **SHP/2** | 4,801 | 100 | 3 | 3,769 | 1,032 | 1,093 | 803 |
| **81TDH2** | 4,824 | 102 | 4 | 3,796 | 1,028 | 1,115 | 820 |

**Table S3: Summary of the plasmids and phage sequences present in genome of sequenced strains and CRISPRs identified**

| **Strain** | **No. of Plasmids** | **No. of pVOGs of Siphoviridae** | **No. of pVOGs of Myoviridae** | **f237 ORF** | **Confirmed CRISPRs identified** | **Putative CRISPRs identified** |
| --- | --- | --- | --- | --- | --- | --- |
| **HP1** | - | 19 | 6 | Present | **0** | **2** |
| **NUK/7** | 4 | - | - | Present | **0** | **1** |
| **VP32** | 2 | - | 20 | Present | **0** | **0** |
| **SHP/2** | 2 | 8 | 30 | Present | **0** | **1** |
| **81TDH2** | - | - | - | - | **0** | **1** |


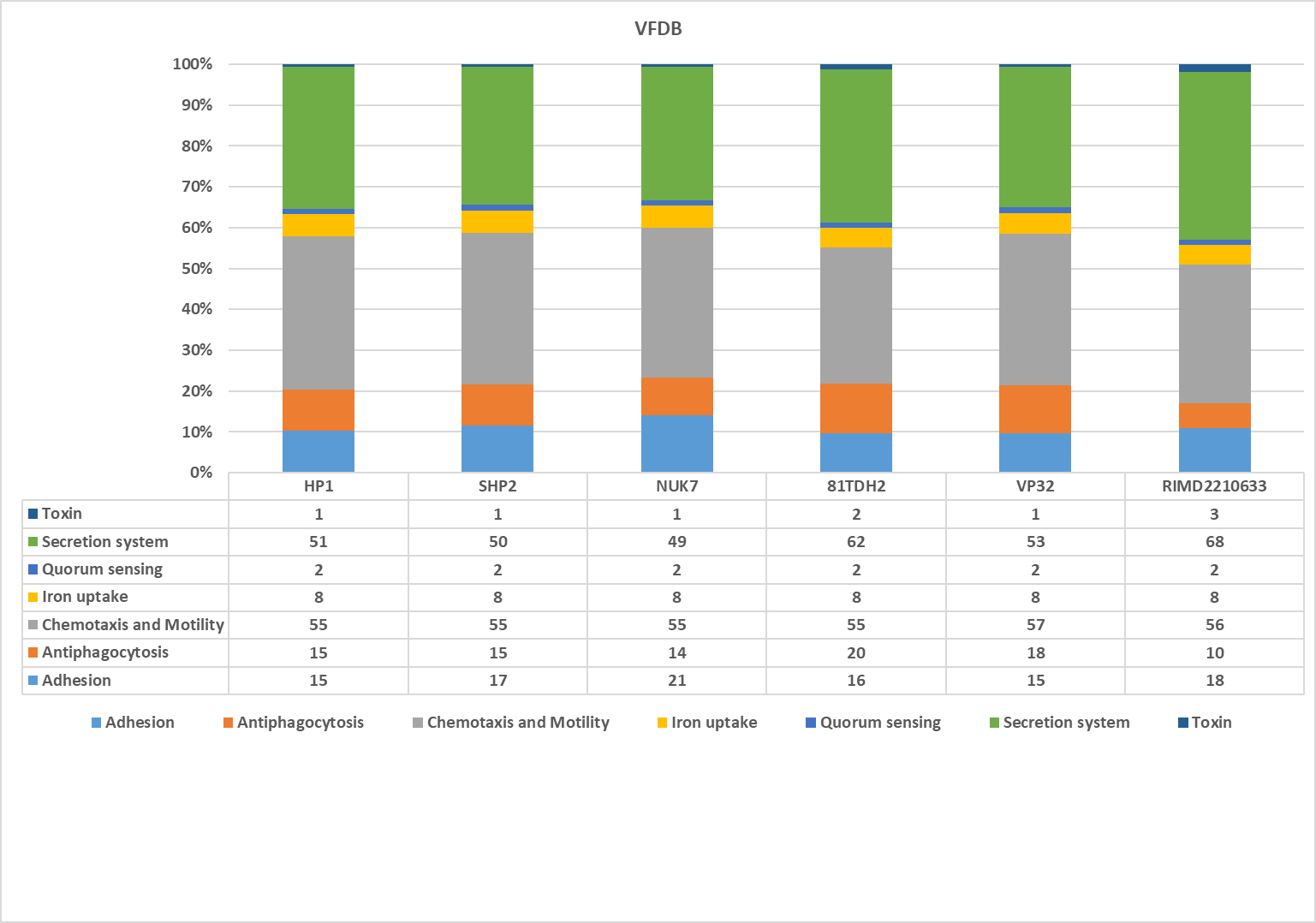


**Figure S1: Number of virulence factor genes per functional category in the genome of sequenced strains analyzed in this study using VFDB database in comparison with reference strain RIMD2210633.**


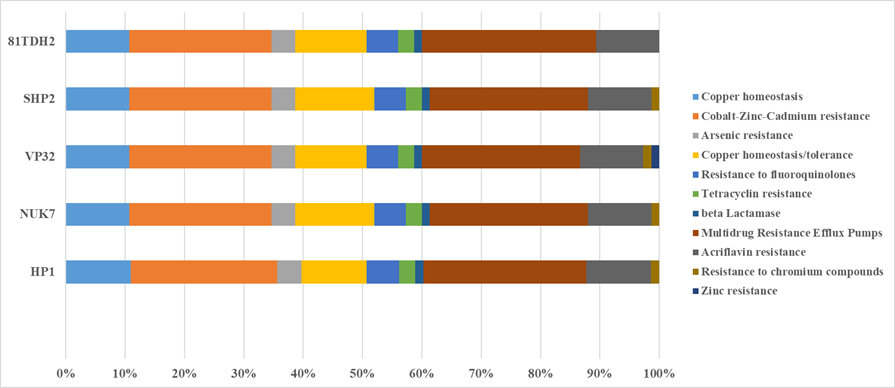


**Figure S2: Antibiotic and heavy metal resistance genes identified using comprehensive analysis with PATRIC server for sequenced strains**
